# Direct bacterial identification from blood culture by matrix-assisted laser desorption-ionization time of flight mass spectrometer using a simplified protocol

**DOI:** 10.1186/cc14069

**Published:** 2014-12-03

**Authors:** A García-Tapia, I Guerrero, T Trujillo, F Galán, P Marín, C Fernández, M Rodriguez-Iglesias

**Affiliations:** 1 Clinical Microbiology Laboratory, Puerta del Mar University Hospital, Cadiz, Spain

## Introduction

Throughout the world, the number of patients at risk for bloodstream infections (BSIs) continues to rise. BSIs are associated with high rates of morbidity and mortality, and they markedly increase the costs of hospital care. Prompt identification of the causative agent(s) and rapid initiation of appropriate antimicrobial therapy are critical for reducing mortality, especially in patients with septic shock. Matrix-assisted laser desorption-ionization time of flight (MALDI-TOF) equipment is increasingly used in the microbiological laboratory. The goal of the present investigation was to apply mass spectrometry directly from positive blood cultures in order to detect and identify bacterial strains using a simplified homemade protocol.

## Methods

One-hundred and sixty-six positive blood cultures (Bactec FX; Becton Dickinson) were analyzed. Gram and conventional plates were used and the identification was done by Wider System (Soria Melguizo). In the MALDI-TOF protocol, positive blood cultures were processed as follow: 5 ml sample of positive broth was extracted and centrifuged at 1,000 rpm for 10 minutes in order to remove blood cells. The supernatant was collected and centrifuged at 4,000 rpm for 15 minutes, then the supernatant was removed and the pellet was placed directly onto a MALDI-TOF target plate and dried at room temperature, subsequently adding 1 μl HCCAMatrix solution (10 mg/ml cyano-4-hydroxycinnamic acid) and drying. Mass spectra were acquired with a Microflex LT mass spectrometer using FlexControl 3.3 software (Bruker Daltonics GmbH).

## Results

One-hundred and forty-nine (89.7%) blood cultures were correctly detected. Thirty-three bacterial species were identified as being *Escherichia coli *(47), the most frequent, followed by *Pseudomonas aeruginosa *(14), *Enterobacter *spp. (11), *Klebsiella *spp. (9), *Staphylococcus aureus *(9), coagulase-negative staphylococci (9), *Enterococcus *spp. (8), *Bacteroides *spp. (7) and streptococci (6). Bacteria fastidious as *Burkholderia, Raoultella, Delftia, Listeria, Acinetobacter *or *Stenotrophomonas *were correctly identified. Only one strain of *Streptococcus pyogenes *and 14 strains of coagulase-negative staphylococci cannot be identified. In hemolyzed samples, it was not possible to identify two strains of *S. aureus*.

## Conclusion

Using MALDI-TOF MS for bacteria identification directly from blood cultures represents an advance in the treatment of septic patients. Compared with conventional culture-based methods, this approach can improve species-level identification of bloodstream isolates in terms of time, accuracy, and costs. We conclude that this technique is simple, fast and reliable for direct bacterial detection from positive blood cultures.

